# Supplementary material for: Family lexicon: Using language models to encode memories of personally familiar and famous people and places in the brain
Source: PLoS One. 2024 Nov 22;19(11):e0291099. doi: 10.1371/journal.pone.0291099 (PMC11584084; doi:10.1371/journal.pone.0291099)
Supplement: S1 File — (PDF) [file pone.0291099.s001.pdf]

S1 Supporting Information

Questionnaire for personally familiar entities (translated from Italian)

People

Name 8 people that you know well and personally, among the people with whom you feel you have a special and positive relationship: people to whom you would turn to to ask for suggestions over personal life choices, people that you meet often, people that you meet and talk to regularly and care about.

Number	Name and surname	Occupation
1		
2		
3		
4		
5		
6		
7		
8		

Imagine having to describe all of these people one by one - e.g. to someone who's never met them - by answering to the following questions. We ask you to provide answers that come to you naturally, and not to overthink them. At the same time, it is important that you provide as many details as possible, both concrete and abstract.

Question 1. How would you describe your relationship with this person?

Question 2. What is this person's occupation in their life?

Question 3. How would you describe this person, both in physical terms and personality?

Question 4. How, where and when did you meet? What did you do?

Question 5. Talk about a recent time you have spent together and that is particularly salient among recent memories.

Question 6. What's the most intense (or dearest) memory of something you have been doing together?

Question 7. Close your eyes and name up to 10 words (both concrete and abstract) that come to your mind when you think about this person.

Question 8. Imagine meeting this person later today, what would you ask them, or talk to them about?

Question 9. Say out loud a sentence that you associate to this person (e.g. their motto), or one of the things they love doing.

Places

Choose 8 places that have a proper name (e.g. a city, a region, a square or a meeting point, a bar, etc.) that you know well and personally, or that you have visited, with whom you feel you have a special and positive relationship, and where you would return to if looking for a place where to feel well.

Number	Name	Type of place
1		
2		
3		
4		
5		
6		
7		
8		

Imagine having to describe all of these places one by one - e.g. to someone who's never met them - by answering to the following questions. We ask you to provide answers that come to you naturally, and not to overthink them. At the same time, it is important that you provide as many details as possible, both concrete and abstract.

1. How and why is this place important to you?
2. What kind of place is it?
3. How would you describe it, both in terms of how it looks and of the kind of emotions it evokes in you?
4. Can you tell us about the first time you were there?
5. What is something you have recently done in that place and that stands out among other memories of that place?
6. What's the most intense (or dearest) memory of something you did there?
7. Close your eyes and name up to 10 words (both concrete and abstract) that come to your mind when you think about this place.
8. Imagine going to this place later today, what would you do (or like to do) there?
9. Say out loud a sentence that you associate to this place (e.g. their motto), or something that captures that spirit of the place.
